# Strength of Excitation Is Negatively Associated with Aggressive Behavior after Interpersonal Rejection

**DOI:** 10.3389/fpsyg.2017.00296

**Published:** 2017-02-28

**Authors:** Joanna Rajchert, Mikołaj Winiewski

**Affiliations:** ^1^Institute of Psychology, The Maria Grzegorzewska UniversityWarsaw, Poland; ^2^Faculty of Psychology, University of WarsawWarsaw, Poland

**Keywords:** temperament, strength of inhibition, strength of excitation, aggressive behavior, interpersonal rejection

## Abstract

This study explored how the Pavlovian temperamental traits strength of excitation (SE) and strength of inhibition (SI) were related to rejection and aggression. We predicted that rejection would increase aggression, but that higher SE and SI would mitigate this effect. Participants (*n* = 117) completed Strelau and Zawadzki’s (1998) Pavlovian Temperament Survey. A week later they were told that a peer wanted (acceptance) or did not want (rejection) to work with them and they were given a chance to react aggressively by damaging that person’s chance of getting a job. We found that only high SE was negatively related to rejected individuals’ aggression. The results are related to the diathesis-stress and catalyst models’ accounts of the role of temperament in shaping experience of social stress.

## Introduction

Interpersonal rejection is a major threat to human well-being and so could be considered an intense stressor. Even moderately intensive interpersonal rejection, such as telling someone that he or she has not been chosen for group work, is followed by symptoms of psychological stress, such as negative emotions (anger, fear, anxiety, distress, and hostility) (Smart Richman and Leary, 2009) and physiological reactions such as the cortical and cardiovascular changes associated with high arousal (Stroud et al., 2000; Gunnar et al., 2003; Blackhart et al., 2007; Ford and Collins, 2010). There is evidence that rejection also leads to aggression in laboratory contexts, where it is variously operationalized as the administration of a higher intensity of noise to the target, the allocation of more hot sauce to the target or a more negative evaluation of an interaction partner which might adversely affect his or her job prospects (e.g., Baumeister et al., 2007; DeWall et al., 2010). The effect of rejection on aggression may, however, be mitigated by particular individual characteristics, such as narcissism, trait self-control, trait-anger, trait-aggressiveness, readiness for aggression and impulsivity (Bushman and Baumeister, 1998; Twenge and Campbell, 2003; Tangney et al., 2004; Bettencourt et al., 2006; Rajchert et al., 2016).

Research (Rajchert and Winiewski, 2016) showed that also temperamental characteristics (strength of Behavioral Activation and Inhibition System) play a role in rejection – aggression relationship. Having a sensitive Behavioral Activation System (BAS) increased displaced aggression after ostracism, whilst having a strong Behavioral Inhibition System (BIS) limited direct aggression (negative evaluation of the interaction partner) after rejection. Other research highlights the role of vulnerability to environmental stressors, expressed in temperamental, physiologic or genetic make-up of individuals (for review see Belsky and Pluess, 2009). Vulnerable individuals tend to react more negatively to adverse conditions, such as neglectful or insensitive parenting (e.g., Caspi et al., 2002) and other negative life events. Moreover, research conducted with infants and toddlers indicates that parental rejection increased externalizing symptoms in children, particularly highly irritable children and children with a difficult temperament (e.g., Kochanska, 1993; Belsky et al., 1998; Morris et al., 2002). The present study adds to the growing literature on individual differences in reactivity to rejection by focusing on experimentally induced rejection and another temperamental traits, namely strength of excitation (SE) and strength of inhibition (SI).

Pavlov’s (1952) theory of the basic propensities of the nervous system - developed further by Strelau et al. (1999) - posits that the central nervous system has two main propensities: SE and SI. SE and SI are theoretical constructs that predict inter-individual variability in the dynamic of behaviors, which is reflected in tempo and intensity of reaction. SE is also referred to as the ‘strength’ or ‘endurance’ of the nervous system, and high SE is characterized by chronically low arousal and low activation even in threatening situations. The stronger the nervous system, the lower the excitation elicited by stressful stimuli. SE is the individual’s ability to endure intense or long-lasting stimulation without passing into protective inhibition (apathy). The second propensity is defined by the ease with which the nervous system creates conditional reactions (extinction, differentiation, and delay) and manifests when it is necessary to adapt a response to environmental requirements.

These temperamental features, that is SE and SI, may inhibit aggressive reactions to rejection in two distinct ways. Endurance (arousability, SE) moderates the state of stress which occurs after rejection whereas inhibition (SI) gives an individual voluntary control over his or her aggressive urges. Developmental studies have shown that high arousability, which is theoretically related to low levels of SE and is also referred to as inefficient involuntary or automatic control is positively associated with aggressive tendencies, aggression and conduct problems in children and adolescents. Whereas effortful control, which is theoretically related to SI and encompasses processes such as attention, activation and inhibitory control is negatively related aggression and antisocial behavior (Capaldi and Rothbart, 1992; Kochanska, 1993; Rothbart et al., 1994; Derryberry and Rothbart, 1997; Eisenberg et al., 2000). In accordance to this, we propose that the temperamental traits SE and SI are independently negatively related to aggressive behavior following rejection.

It should be noted that part of the data used in the second study described by Rajchert and Winiewski (2016) were also used in this study, namely index of aggression after rejection, although with reference to different moderators of the rejection - aggression relationship.

## Materials and Methods

### Participants

Data from 117 first-year pedagogy and psychology students (102 women and 15 men), aged from 19 to 24 years (*M* = 19.5, *SD* = 0.95) were included in analysis. The data from 23 other participants were dropped from the sample because they indicated the disbelief in the cover story of rejection manipulation or the aggression measurement. Students were told that participation in the study would allow them “to get to know how the psychological studies look like and help them to design their own studies in the future.” This was the only encouragement they received to participate. Students were not given grades or points for participation and were allowed to withdraw from participation in the study at any time for any reason. All students agreed to participate and none of them withdrew. The sex distribution in the sample was typical of the population of social science students. The study was carried out in accordance with the recommendations of Academic Ethical Review Board and all participants provided written, informed consent in accordance with the Declaration of Helsinki.

### Measures

#### Measurement of Strength of Excitation and Strength of Inhibition

Participants completed the two scales from the Polish version of the Pavlovian Temperamental Survey (PTS; Strelau et al., 1999; Polish version by Strelau and Zawadzki, 1998) that measure SE and SI. Both scales consist of 19 items to which responses are given using a four-point Likert scale ranging from 1 (completely agree) to 4 (completely disagree). The original Polish version of the questionnaire has good internal validity (Cronbach’s alpha was 0.75 for SI and 0.85 for SE) and reliability. Ruch et al. (1991) showed that PTS variables correlated with the corresponding dimensions of Eysenck’s Personality Questionnaire (Eysenck et al., 1985): SE was positively correlated with extraversion and negatively correlated with neuroticism, whereas SI was negatively correlated with psychoticism, neuroticism and extraversion.

#### Aggressive Behavior Measurement

We measured aggressive behavior using a negative evaluation procedure similar to that used in other studies (Rothaus and Worchel, 1960; Wingrove and Bond, 1998; Twenge et al., 2001); negative evaluation has been recognized as a valid operationalization of aggressive behavior (Bettencourt et al., 2006). The experimenter told the participant that the peer who had assessed his or her video was applying for a job as the experimenter’s assistant. To apply, she needed an assessment from her peers, which the participant could provide. All participants agreed and completed a 10-items questionnaire about her suitability for the job (e.g., ‘You can rely on this person’; ‘This person is friendly’) using a 10-point Likert scale ranging from 1 (*completely disagree*) to 10 (*completely agree*). The scores were reverse coded before being summed so that the index would represent a negative evaluation.

### Procedure

The first part of the study was conducted during an introductory psychology class and presented as a separate activity (not connected to the later experimental procedure). The students completed the PTS and the BIS/BAS scale (Carver and White, 1994). The results of analyses of BIS/BAS scales as moderators of the association between rejection and aggression were described by Rajchert and Winiewski (2016) and so they are not presented here. One week after they had completed the assessment of temperament the same students were individually invited to participate in a study that was ostensibly about ‘social networks and social media.’ The recruitment procedure of volunteers was the same as the procedure of questionnaires completion. All participants who completed the PTS scale agreed to take part in the ostensibly second study and signed separate informed consent. During the subsequent procedure we induced a feeling of rejection or acceptance and measured aggressive behavior.

#### Manipulation of Rejection

The procedure was inspired by Twenge et al. (2001), who showed that social exclusion caused people to retaliate aggressively toward those who had excluded them. Similar procedures have been used in other studies of exclusion (e.g., Leary et al., 1995; Nezlek et al., 1997). Participants were told that they had been randomly paired with another participant, a ‘peer’ (actually a female confederate) whom they would never meet in person, although they would get to know each other through a video interview. First, participants watched a video clip of the female confederate answering questions such as ‘What is your favorite food in the cafeteria?’ All participants watched the same pre-recorded video. The participants were asked to rate the interview and decide whether they would like to work with the person who had appeared in the video. After participants had done this and their ratings had been collected they were video-recorded answering similar questions. Their feedback and video was delivered to the female peer by the experimenter, who reassured participants that the peer would watch and rate their video before receiving the feedback they had given on her interview (we did not want participants to think that ratings made by the peer had been biased by reading their feedback). When the experimenter returned participants were told whether their ‘peer’ had liked their interview and wanted to work with them in the future. Participants were randomly assigned to experimental conditions. In the rejection condition (*n* = 56) the feedback was: ‘The interview was poor! I would not like to work with this person’ and in the acceptance condition (*n* = 61) it was ‘The interview was cool. I would like to work with this person.’ After this information the aggressive behavior was measured and all participants were debriefed.

## Results

First, we calculated zero-order correlations (**Table 1**). Point-biserial correlations indicated that there was a strong association between rejection (experimental condition) and aggressive behavior. In the rejection condition, responses to the partner were substantially more aggressive (this is the only result described in Rajchert and Winiewski, 2016; all other results presented here are original and have not been presented elsewhere). SE was negatively related to aggression and men had higher SE scores.

**Table 1:** Zero-order correlations.

	1	2	3	4
(1) Aggressive behavior				
(2) Strength of excitation (SE)	-0.22^∗^			
(3) Strength of inhibition (SI)	-0.09	0.14		
(4) Gender (0 – female)	-10	0.21^∗^	-0.05	
(5) Rejection (0 – acceptance)	0.61^∗∗^	0.02	-0.13	-0.06^a^
*M*	4.66	2.32	2.64	
*SD*	2.09	0.45	0.38	
Minimum	1	1.47	1.27	
Maximum	10	3.58	3.47	
Cronbach’s alpha	0.97	0.86	0.71	

*^∗^*p* < 0.05, ^∗∗^*p* < 0.001; ^a^Phi coefficient*.

We used hierarchical multiple regression to investigate the impact of social rejection on aggressive behavior and the moderating role of Pavlovian temperamental features (SI and SE); the two interaction terms (experimental condition with SE and SI) were introduced in the second step (**Table 2**).

**Table 2:** Hierarchical multiple regressions models with Pavlovian temperamental dimensions as predictors of aggressive behavior.

	Step 1		Step 2	
	*B*	*SE B*	*B*	*SE B*
Constant	3.43^∗∗∗^	0.22	3.45^∗∗∗^	0.21
Rejection (0 – acceptance)	2.59^∗∗∗^	0.30	2.60^∗∗∗^	0.29
SE	-1.07^∗∗^	0.35	-0.05	0.49
SI	0.13	0.40	0.34	0.79
Gender (0 – female)	-0.12	0.46	-0.23	0.44
SI × Rejection			-0.31	1.02
SE × Rejection			-1.84^∗∗^	0.67
*R^2^*	0.43		0.48	
*F*	21.23^∗∗∗^		16.33^∗∗∗^	
Δ*R^2^*			0.04	
Δ*F*			4.14^∗^	

*^∗^*p* < 0.05, ^∗∗^*p* < 0.01, ^∗∗∗^*p* < 0.001*.

**FIGURE 1:**
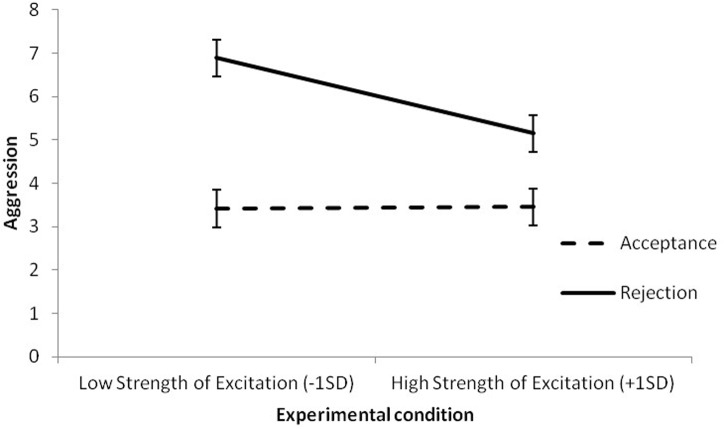
**Interaction effect of rejection and strength of excitation on aggression**. Slopes are plotted at the ±1 *SD*.

Analyses showed that, after controlling for rejection - which was the strongest predictor of aggressive behavior - SE was negatively related to aggressive reaction. The interaction terms were added in the next step, which showed that only the interaction of SE with rejection explained aggressive reaction. To explain the interaction we calculated a simple slope for high and low (±*SD*) values of SE. Analyses revealed that the effect of rejection on aggression was high and negative for low values of SE (*B* = 3.47, *SE* = 0.43, *p* < 0.001) but was smaller at high values of SE (*B* = 1.69, *SE* = 0.42, *p* < 0.001) (see **Figure 1**). The interaction shows that in high SE participants, social exclusion prompted a less aggressive response toward a peer who had rejected them earlier than it did in low SE participants.

## Discussion

Numerous studies have indicated that rejection or exclusion conditions cause more aggressive behavior than an acceptance condition (for review, see Leary et al., 2006; Baumeister et al., 2007), especially when rejected individuals have no chance of regaining their included status. Our study provided further confirmation of this finding as rejected participants were more aggressive than accepted participants (see also Rajchert and Winiewski, 2016).

There are also studies showing that individual differences influence the magnitude of the aggressive response to rejection (Twenge and Campbell, 2003; Buckley et al., 2004; Ayduk et al., 2008; Rajchert, 2015; Rajchert et al., 2016). However, although the role of temperament in response to stress and coping behaviors is well-recognized, we are not aware of any research that has looked at the temperamental features that moderate the rejection–aggression association other than recently published results from the same study which we discuss here (Rajchert and Winiewski, 2016) dealing with how BIS/BAS strength influences the rejection–aggression relationship. This research explored the role played by two temperamental traits, SE and SI, in the relationship between rejection and direct, retaliatory aggression. Higher central nervous system strength (SE) - manifested as the ability to function effectively even during intense stimulation - contributed to lower aggression toward a partner after rejection. We also observed a main effect of SE, with higher SE resulting in less aggression, when controlling for rejection. We conclude, like Chess and Thomas (1991) and Strelau (1995), that high SE participants either experienced the rejection episode as less stressful or were able to cope with the situation better than individuals low on SE. The exact mechanism through which SE reduces aggression remains to be determined. At this point we can only cite research on correlates of SE which showed that arousability, which is the foundation of SE, is also related to other lower-order temperamental traits such as emotional reactivity, positive and negative affect, behavioral activation and inhibition systems, neuroticism and introversion (Strelau, 1994). Most of these features have, in turn, been related to the perceived intensity of stress and to coping responses (e.g., Watson et al., 1988; Heponiemi et al., 2003; Strelau and Zawadzki, 2005; Boyes and French, 2009). Our study adds to these results by showing that a common neurophysiologically and genetically grounded feature, namely arousability (manifested as SE) may underlie many of the findings linking personality variables with reductions in aggressive, retaliatory responses to rejection.

We also predicted that high inhibition would restrain aggressive behavior after exclusion, but this prediction was not fulfilled. The most plausible explanation for this is that participants in our experiment perceived retaliatory aggression as justified and fair under the circumstances and therefore did not inhibit their aggression. SI represents the capacity to delay or refrain from action when it is considered appropriate to do so. It is not a blanket tendency to inhibit impulses. In Pavlov’s theory and experiments, SI was defined as the ease with which dogs learned conditional reactions and in this sense inhibition is observable only in particular situations. In our experiment the partner evaluated rejected individuals very harshly and wrongfully on the basis of scant evidence; it is therefore unsurprising that when given the opportunity, they reciprocated. Other research has shown that when individuals are convinced that rejection is unfair and undeserved it elicits a more intense aggressive reaction (Smart Richman and Leary, 2009). The second explanation is that participants’ negative evaluation of the target who had rejected them, although aggressive toward the target, could be perceived as prosocial in the sense that it potentially protected other people from being exposed to an experimenter with a tendency to unwarranted insulting behavior. The third explanation refers to the I^3^ theory. Denson et al. (2012) explained aggressive responses as the sum of the interaction of three components: self-regulatory strength (Inhibition), provocation strength (Instigation) and Impellance. This would imply that in our experiment the instigation to aggression was not fully counterbalanced fully by the inhibitory factors.

## Conclusion

The following limitations should be taken into consideration when interpreting the results of the study. First of all, the majority of the sample was female. Studies show that there are sex differences in direct, physical aggression in real-world settings (Archer, 2004). There has been much less research on the issue of sex differences in the context of laboratory studies of the rejection – aggression relationship, which measure mild forms of aggression. Other studies which have used similar rejection manipulations and measures of aggression did not even analyze sex differences (Twenge et al., 2001; Buckley et al., 2004). We controlled for the effects of sex statistically, nevertheless our results should be replicated in a sample with a more even sex distribution, possibly not made up of college students.

Another potential limitation is the use of peer evaluation as an index of aggression. Although this measure has been used in other studies of aggressive behavior (e.g., Twenge et al., 2001) it is open to criticism. Anderson and Bushman’s (2002) definition of aggression specifies that the target of aggression must be motivated to avoid being hurt and the aggressor must be motivated to hurt the target and must be convinced that the intended behavior will hurt the target. It is conceivable that the peer evaluation might not be considered hurtful by the target and there are various motivations for giving a negative evaluation other than aggression (for example providing reliable feedback on the interaction partner to the future employer). In future studies other measures of aggression should be used to check whether the interaction effect is robust.

In summary, our study shows that although rejection contributes to aggressive behavior, the intensity of the aggression elicited by regression also depends on core temperamental traits. A very basic trait, the strength of nervous system, which, for example, determines an individual’s capacity to work effectively in over-stimulating environments or dangerous situations, also influences how bearable rejection is. On the other hand, the a strong inhibitory capacity, which manifests in the covering of feelings and the ability to delay or refrain from initiating behavior, does not always diminish aggression. In some instances, behaving aggressively might serve a higher cause or be regarded as appropriate, for example aggression intended to ‘teach someone a lesson’ or to protect others (Ferguson and Beaver, 2009). In such cases one would not necessarily observe an angry outburst of aggression related to low self-regulation; one might also (or instead) observe a cool, intentional act of aggression.

## Ethics Statement

This study was carried out in accordance with the recommendations of the Academic Ethical Review Board (Scientific Research Ethics Committee of The Maria Grzegorzewska University). All subjects gave written informed consent in accordance with the Declaration of Helsinki.

## Author Contributions

JR prepared the manuscript (wrote introduction, method and discussion, formated and submitted), planned and conducted the research, interpreted the results. MW analyzed the data prepared the result section of the manuscript; he also took part in planning the content of manuscript parts and in interpretation of results.

## Conflict of Interest Statement

The authors declare that the research was conducted in the absence of any commercial or financial relationships that could be construed as a potential conflict of interest.
